# Preparation and Deep Characterization of Composite/Hybrid Multi-Scale and Multi-Domain Polymeric Microparticles

**DOI:** 10.3390/ma12233921

**Published:** 2019-11-27

**Authors:** Wei Yu, Nikunjkumar Visaveliya, Christophe A. Serra, J. Michael Köhler, Shukai Ding, Michel Bouquey, René Muller, Marc Schmutz, Isabelle Kraus

**Affiliations:** 1 Université de Strasbourg, CNRS, ICS UPR 22, F-67000 Strasbourg, France; wei.yu@etu.unistra.fr (W.Y.); ca.serra@unistra.fr (C.A.S.); shukai.ding@etu.unistra.fr (S.D.); michel.bouquey@unistra.fr (M.B.); rene.muller@unistra.fr (R.M.); marc.schmutz@ics-cnrs.unistra.fr (M.S.); 2 Department of Physical, Chemistry and Microreaction, Technology, Technical University of Ilmenau, 98694 Ilmenau, Germany; nvisaveliya@ccny.cuny.edu (N.V.); michael.koehler@tu-ilmenau.de (J.M.K.); 3 Shaanxi University of Science & Technology, Institute of Atomic and Molecular Science, Xi’an 710021, China; 4 Université de Strasbourg, CNRS, IPCMS UMR 7504, F-67000 Strasbourg, France

**Keywords:** microfluidics, nanoemulsion, polymer microparticle, gold nanoparticles, silver nanoparticles, polymer nanoparticles, composite, hybrid

## Abstract

Polymeric microparticles were produced following a three-step procedure involving (i) the production of an aqueous nanoemulsion of tri and monofunctional acrylate-based monomers droplets by an elongational-flow microemulsifier, (ii) the production of a nanosuspension upon the continuous-flow UV-initiated miniemulsion polymerization of the above nanoemulsion and (iii) the production of core-shell polymeric microparticles by means of a microfluidic capillaries-based double droplets generator; the core phase was composed of the above nanosuspension admixed with a water-soluble monomer and gold salt, the shell phase comprised a trifunctional monomer, diethylene glycol and a silver salt; both phases were photopolymerized on-the-fly upon droplet formation. Resulting microparticles were extensively analyzed by energy dispersive X-rays spectrometry and scanning electron microscopy to reveal the core-shell morphology, the presence of silver nanoparticles in the shell, organic nanoparticles in the core but failed to reveal the presence of the gold nanoparticles in the core presumably due to their too small size (c.a. 2.5 nm). Nevertheless, the reddish appearance of the as such prepared polymer microparticles emphasized that this three-step procedure allowed the easy elaboration of composite/hybrid multi-scale and multi-domain polymeric microparticles.

## 1. Introduction

Microfluidics is the science and technology of systems that process or manipulate small amounts of fluids (10^−9^–10^−18^ L) using microchannels with dimensions of tens to hundreds of micrometers [1]. It is an interdisciplinary field that has largely contributed to the development of flow chemistries [2]. Use of microfluidic devices allowed us to explore the possibility to run chemical reactions in new operating windows (higher T, pressure and reactant concentrations) [3] and to produce organic and inorganic materials with better defined or new properties [4,5].

Specifically, microfluidic systems have shown the possibility to allow synthesizing and assembling polymeric microparticles with narrow size distribution and various sizes, shapes, morphologies and compositions [6,7,8,9]. Such polymeric microparticles are usually obtained from monomer-based microdroplet by either thermal-induced or UV-initiated polymerization. Indeed, microfluidic devices are extremely efficient emulsification systems, which allow us to produce either oil-in-water (o/w) or water-in-oil (w/o) size-controlled macroemulsions. Moreover the droplet size distribution is usually extremely narrow as its coefficient of variation is typically lower than 5% [8]. Photopolymerization is usually preferred over thermal-induced polymerization for a couple of reasons: (i) this a quite efficient method for converting the liquid monomer droplet into a solid polymer material within few tens of seconds [10] and (ii) it can easily be implemented in flow and such can fix rapidly non-thermodynamically stable droplet morphologies before relaxing phenomena can occur.

Two different categories of microfluidic devices have been reported for the emulsification of a polymerizable liquid [9]. In the first one both continuous and dispersed fluids flow inside microchannels while in the second one the continuous phase flow inside a tube and the dispersed phase inside a capillary of small dimensions. The emulsification mechanism, which is quite similar for these two categories, proceeds from the break-up of a liquid thread into droplets when the to-be dispersed phase is sheared by the continuous and immiscible phase. Three microchannel-based devices are commonly found: the terrace-like microchannel device, the T-junction microchannel device and the flow focusing microchannel device (FFD). The terrace-like microchannel device [11,12] consists of a main channel in which flows the continuous phase. Several microchannels deliver the dispersed phase at the top and from both sides of the main channel. Then terraces located just below the microchannels allow the break-up of the dispersed phase thread. In T-junction microchannel devices [13,14], the to-be dispersed phase is delivered through a microchannel perpendicular to a main channel in which flows the continuous phase. Depending on the flow rates of the continuous and dispersed phase, the break-up is observed at the junction of the two microchannels or further downstream. Flow focusing devices (FFDs) are based on the principle of hydrodynamic focusing [15]. The dispersed phase flows in a central microchannel while the continuous phase is delivered through two side channels. In front of the central channel, a small orifice or a restriction allows the continuous phase to pinch the dispersed liquid thread, which breaks pass the orifice into droplets. Three capillary-based devices are also commonly found: the co-flow capillary device, the cross-flow capillary device and the flow-focusing capillary device. The characteristic of the co-flow device is that the dispersed phase flows in the same direction as the continuous phase flow [16,17]. In the cross-flow device the dispersed phase flows in a direction perpendicular to that of the continuous phase flow like for the T-junction microchannel device [18]. The characteristic of the flow-focusing device is that the dispersed phase flow is axisymmetrically pinched by the flow of the continuous phase resulting in the elongation of the dispersed phase stream, which breaks up into droplets under capillary instabilities [19]. Capillary-based devices has the following advantage over microchannel-based devices: the dispersed phase is flowing in the centerline of the continuous phase and thus could be kept away from the device wall where phase inversion may happen in case the wettability of the dispersed phase is greater than that of the continuous phase. Hence, a single capillary-based devices can produce o/w or w/o emulsions. However, they do have some drawbacks too: they are somehow limited in the flow arrangement and design. Although each device from these two categories has its own characteristics, some general trends can be drawn for the control of the particle size [6,20,21]. Two dimensionless numbers, for each phase, were identified, which significantly contribute to the final particle diameter: the Reynolds number (Re) and the capillary number (Ca), which mainly depend upon the fluids density and viscosity, the mean fluids velocity, a characteristic dimension of the flow (usually the channel width) and the interfacial tension between the two immiscible fluids [22,23]. Thus, for a given formulation of dispersed and continuous phases, the particle diameter is primarily a function of the velocities or flow rates of the two phases. Generally, an increase in the continuous phase flow rate or a decrease in the dispersed phase flow rate induces a decrease of the mean polymer particle diameter. This diameter is also affected by the interfacial tension; lower interfacial tensions leading to smaller particles. Fluids viscosity plays as well an important role. An increase in the continuous phase viscosity or a decrease in that of the dispersed phase is usually followed by a decrease in the final particle size. Finally, it was observed that a reduction in the characteristic dimension of the microsystem, e.g., the channel width for the terrace-like microchannel and T-junction devices, the orifice width for the FFD and the inner capillary diameter for the capillary-based devices, generates smaller particles.

Besides the simple droplet morphology, double droplets, i.e., a droplet in another droplet, can be produced by slight modifications of the aforementioned FFD and a co-flow capillary-based device. Thus FFD was modified to accommodate two additional side microchannels [24] so as to deliver at the same time three different phases: the inner channel delivering the embedded droplet phase, the middle microchannels the main droplet phase and the outer phase the continuous phase. The same can be obtained with two co-axially arranged capillaries [25]. Upon polymerization, these double droplets led to the formation of core-sell polymer microparticles whose core and shell sizes can readily be tuned upon varying the flow rates of the three phases.

Microfluidic devices not only present advantages to prepare microscale particles but also nanoscale ones. Recently, preparation of polymeric nanoparticles has been explored using microfluidic devices to control the size [26], morphology [27] and shape [28] of the produced nanoparticles. Two methods were mostly developed depending whether a monomer phase or a polymer phase was used as the starting material. The first method is much comparable to the aforementioned method to produce polymeric microparticles. By applying a specific microfluidic device, the monomer phase is emulsified into nanodroplets, which are later polymerized. One example of such a device was reported by Koehler et al. [29] they fabricated a microhole plate containing 16 holes × 48 holes of 20 µm in diameter by photolithographic techniques. The monomer nanodroplets were generated by the shear forces caused by the tangential flow of a continuous phase at the surface of a microhole plate through which the dispersed phase was pumped. The second method relies on the so-called solvent displacement method in which a polymer precipitates when its solvent diffuses rapidly in a non-solvent of the polymer. The key point in this method to obtained size-controlled polymer nanoparticles is to promote an extremely fast diffusion of the solvent such as to avoid aggregation and low nanoparticles sizes (down to less than 100 nm). This can be achieved either by producing microdroplets of the polymer solution in a stream of polymer non-solvent by means of microchannel based devices [30], or by mixing the polymer solvent with its non-solvent in a microstructured mixer such as to produce a staggered stream of the two solutions for with lamellae thickness can be as low as 45 µm [31].

On the other hand, noble metal nanoparticles (NPs) are increasingly considered in many different fields since they present for example unpreceded electrical, optical and catalytic properties, especially in the fabrication of semiconductor [32]. Energy consumption and pollution are the main challenging issues of this century. UV-irradiation methods are considered as promising techniques for reducing energy consumption and pollutants remediation [33,34]. They were known for long to be an effective method to cure polymeric resins. There were also used for the on-fly polymerization of microfluidic-generated droplets to produce polymeric microparticles having different sizes, shapes and morphologies [9] and for the photoreduction of metal salts [35].

If microfluidic polymer micro and nanoparticles have already found separately applications in many fields such as drug release [36,37,38], sensorics [39] to name a few, they were scarcely combined to produce new materials like composite/hybrid multi-scale and multi-domain polymeric microparticles. In such objects, polymer nanoparticles and possibly inorganic nanoparticles are selectively embedded into different domains of a polymeric microparticle. These materials could have strong applications in fields such as optical sensors and theranostics.

In this paper, we present a facile microfluidic route to the preparation of core-shell polymer microparticles selectively doped with polymer and metal nanoparticles by following a three-step procedure. Morphological structure and chemical Z-contrast (COMPO) of the microparticles were extensively characterized by scanning electron microscope (SEM) and energy dispersive X-rays spectrometry (EDXS) respectively.

## 2. Experimental Section

### 2.1. Materials

Five components were used to prepare a polymerizable o/w nanoemulsion: monomer (15 vol.%), Ostwald ripening inhibitor (4 wt.%/monomer), surfactant (2.5 wt.%/monomer), distilled water (85 vol.%) as well as the organic-soluble photoinitiator (2.5 wt.%/monomer). Specifically, the oil phase was composed of a mixture containing the same weight amount of methyl methacrylate (MMA, 99% purity, supplied by Aldrich) and tri (propylene glycol) diacrylate (TPGDA, supplied by Aldrich), hexadecane (HD, 99% purity, supplied by Sigma Aldrich) as the Ostwald ripening inhibitor and the photoinitiator 1-hydroxycyclohexyl phenyl ketone (HCPK, 99% purity, supplied by Aldrich). Note that TPGDA also acted as a crosslinker due to its two double bonds. The water phase comprised distilled water and sodium dodecyl sulfate (SDS, 95% purity, supplied by Sigma Aldrich) as a surfactant. The double droplets core phase was composed of the above poly(TPGDA-co-MMA) colloidal suspension admixed with a water-soluble photoinitiator (Genocure DMHA, supplied by Rahn, 5 wt.%/water-soluble monomer), a hydrophilic monomer (Acrylamide, 97% purity, supplied by Aldrich, 40 wt.%/water in the colloidal suspension), a crosslinker (N,N′-methylene-bisacrylamide, MBA, 99% purity, 10 wt.%/water-soluble monomer, supplied by Sigma Aldrich) and Tetrachloroauric acid trihydrate (AuHCl_4_·3H_2_O, ≥99.5%, supplied by Roth, 0.17 wt.%/colloidal suspension). The double droplets shell phase was composed of pure TPGDA, span80 surfactant (3.5 wt.%/TPGDA, supplied by Fluka), HCPK (99% purity, 3.5 wt.%/TPGDA, supplied by Aldrich), diethylene glycol (DEG, 99% purity, 99%, 10 vol.%/monomer, supplied by Acros) and silver nitrate salt (AgNO_3_, 0.17 wt.%/monomer, supplied by VWR). Finally, the fluid during the synthesis of core-shell microparticles was composed of an aqueous solution of 2 wt.% methyl cellulose (supplied by Alfa Aesar). Ascorbic acid (supplied by VWR) and silver nitrate were used for the silver enforcement. Distilled water was used throughout the experiment.

#### 2.1.1. First Step: Preparation of the Polymerizable Nanoemulsion

**Figure 1:**
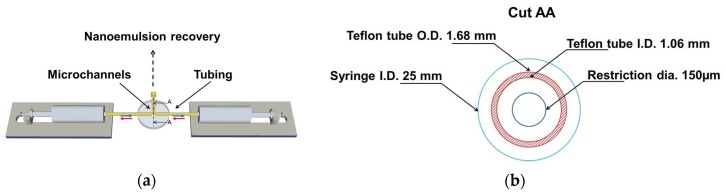
Schematic drawing of the microprocess to prepare the polymerizable nanoemulsion (not at scale, (**a**)), dimensions of the different channels in cut AA (**b**).

Figure 1 depicts the schematic drawing of the elongational-flow microprocess used to produce the polymerizable nanoemulsion. It mainly consisted in two mid pressure syringe pumps (neMESYS Mid Pressure Module, Cetoni), two 25 mL stainless steel syringes and one PEEK tee (Valco Vici) as the elongational-flow microemulsifier/micromixer. The syringe pumps were controlled by the manufacturer’s software and were forced to operate in opposite phase (withdraw/infuse) so as to induce a reciprocating flow rate (RFR) of 20 mL/min through the micromixer. A back and forth movement of the pumps counts for one cycle. The micromixer consisted in three drilled microchannels having a bore size of 150 μm. Two of these microchannels were connected to the two 25 mL stainless steel syringes (Cetoni) by two PTFE tubing (1.06 mm ID × 1.68 mm OD). The third microchannel was used to recover the nanoemulsion once the operation was finished. The TPGDA/MMA oil and water phases composing the nanoemulsion were directly charged without premix into one stainless steel syringe for a total volume of 5 mL. Then, the pumps were switched on. After 200 cycles, one pump was switched off in its zero volume position while the second one pumped the freshly prepared nanoemulsion (a milky-like solution) through the recovery microchannel where it was collected and ready for the characterization and second step. Thus, the obtained nanoemulsion was analyzed by dynamic light scattering (DLS, Zetasizer, Nano Series, Malvern) with a fixed scattering angle of 173° to determine the nanodroplets’ diameter and their polydispersity in size (PDI). It is commonly admitted that nanoemulsions with PDI values below 0.2 can be considered as monomodal.

#### 2.1.2. Second Step: Preparation of the Colloidal Suspension Derived from the Polymerizable Nanoemulsion

**Figure 2:**
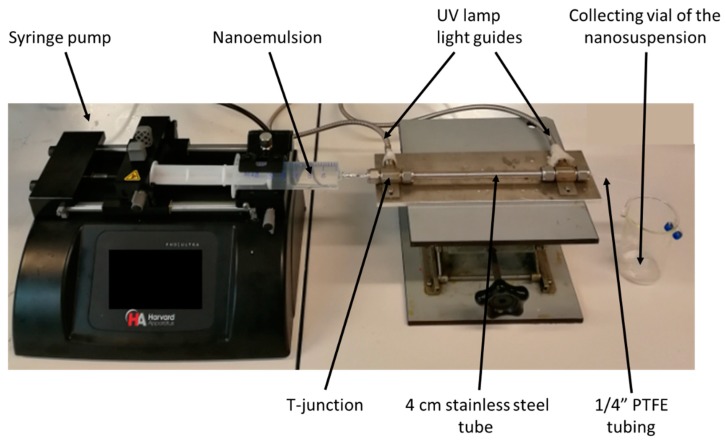
Picture of the UV-initiated polymerization setup to convert the polymerizable nanoemulsion into a colloidal suspension of polymer nanoparticles.

The freshly prepared nanoemulsion was polymerized by UV irradiation in a tube-in-tube device shown in Figure 2. Of the above nanoemulsion 4 mL was charged in a 5 mL plastic syringe (HSW). Then the syringe was placed in the holder of a syringe pump (PHD 2000, Harvard Apparatus) aiming at delivering the nanoemulsion through a 22 cm long PTFE tubing (1.68 mm ID × 3.2 mm OD) at a constant flowrate of 0.025 mL/min. The PTFE tubing was inserted inside a 4 cm ID stainless steel tube equipped at both ends with a T-junction (Swagelok) in which was set the two light guides of an UV source (Lightningcure LC8, Hamamatsu) operating at λ = 365 nm; a wavelength corresponding to the maximum of absorbance of the photoinitiator added into the nanoemulsion monomer phase. Given the size of the PTFE tubing and the flow rate, the residence time of the nanoemulsion under the UV irradiation was about 20 min. At the end of the PTFE tubing, the polymerized nanoemulsion, i.e., colloidal suspension of polymer nanoparticles, was collected in a vial.

#### 2.1.3. Third Step: Production of Core-Shell Microparticles by a Co-Axial Capillaries-Based Microfluidic Droplet Generator

**Figure 3:**
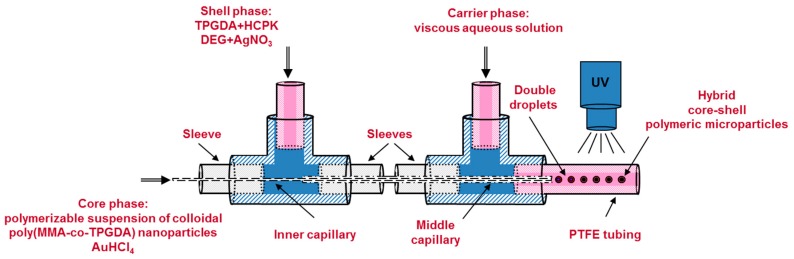
Schematic drawing of the co-axial capillaries-based microfluidic droplet generator for the production of double droplets that were hardened downstream into core-shell microparticles by UV-initiated polymerization.

Acrylamide, MBA, Genocure DMHA and gold salt were first added step by step to the freshly prepared nanosuspension under vigorous agitation to form the core phase of the core-shell microparticles. Then, the mixture was left stirred for an additional 10 min protected from ambient UV light by aluminum foil in order to prevent unwanted reduction of the gold salt. Meanwhile, the shell phase was prepared by dissolving the silver salt in DEG and pouring the resulting solution in a mixture of span 80, HCPK and TPGDA. The carrier phase was prepared separately by dissolving the methyl cellulose in distilled water. Then all three aforementioned phases were injected in the co-axial capillaries-based microfluidic droplet generator of Figure 3 by means of three distinct syringe pumps (PHD 2000, Harvard Apparatus) operating at the following flow rates: Q_core_ = 1.5 μL/min, Q_shell_ = 4 μL/min, Q_carrier_ = 130 μL/min. The droplet generator consisted in a set of two 1/16” T-junction (Upchurch Scientific) and two co-axially arranged fused silica capillaries (Polymicro Technologies). The inner capillary (100 µm ID × 165 μm OD) served to inject the core phase while the middle capillary (530 µm ID × 670 μm OD) allowed delivering the shell phase. Both capillaries tips were placed in the centerline of another PTFE tubing (1.68 mm ID × 3.2 mm OD). With such dimensions and flow rates, and by considering the hydraulic diameters for the shell and carrier phases (365 and 1010 µm respectively), Reynolds numbers for the three flows were calculated to be lower than 1 indicating creeping flows. Upon contact at the tips of the two capillaries, the three phases gave rise to the production of core-shell microdroplets. The formation of such double droplets and methods to make variation of overall droplet and core sizes are reported in a previous paper [24]. Two centimeters downstream the double droplet formation, the same UV-arrangement used in second step was utilized to polymerize the microdroplets into core-shell microparticles and to reduce the metal salts. Given the flow rate of the carrier fluid and length of the UV-arrangement (22 cm), the residence time of a droplet under UV light was about 4 s. At the end of the UV-arrangement, the microparticles were collected into a beaker and washed with distilled water three times to remove the excess methyl cellulose. Then a filter paper was used to separate the microparticles from the aqueous solution. Finally, the microparticles were allowed to dry overnight at room temperature.

### 2.2. Silver Reinforcement

**Figure 4:**
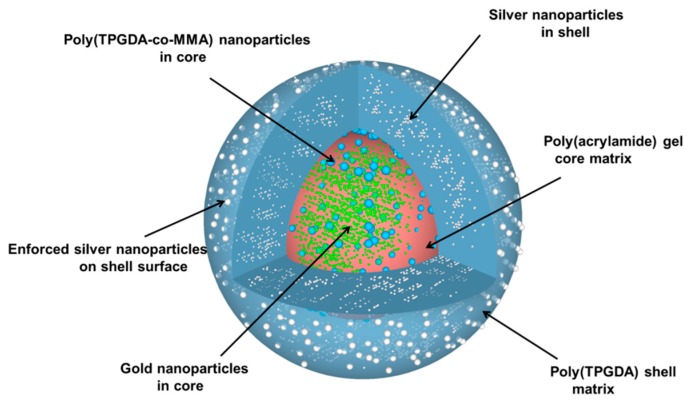
Schematic drawing of a targeted core-shell polymeric microparticle doped with organic and inorganic nanoparticles.

For the silver reinforcement experiment, two different aqueous solutions were prepared; the first one contained 3 mmol/L of ascorbic acid and the second one 2 mmol/L of silver nitrate. Then few core-shell microparticles were immersed into 50 μL of the first aqueous solution in a vial. Next, 50 μL of the silver nitrate aqueous solution was poured into the vial. The reduction of the silver salt was allowed for a few minutes under gentle stirring. Finally, the core-shell microparticles were recovered by paper filtration and allowed to dry overnight at room temperature. Upon all these different steps, the particle morphology that was targeted is represented in Figure 4.

### 2.3. Characterization

Composite/hybrid microparticles were observed by scanning electron microscopy (S4800, SU8010 Hitachi and JSM-6380, JEOL) to characterize their surface and inner structure [40]. For the latter, a single microparticle was fixed in a specific holder from Leica and trimmed with a ultramicrotom (UCT6 Leica) equipped with a cryotrim diamond knife (Diatome) to have a flat surface rendering the core-shell structure visible and EDX analysis possible. SEM secondary electron images and back-scattered electrons images in chemical Z-contrast (COMPO) were recorded (JEOL 6700F) and energy dispersive X-rays spectrometry (EDXS; Thermo-Noran Vantage) was performed to identify the chemical composition.

## 3. Results and Discussion

**Figure 5:**
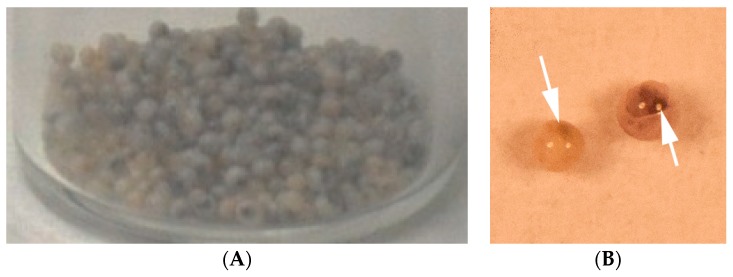
Optical image of dried composite/hybrid core-shell microparticles (**A**) and macro photography of two microparticles, the arrow point towards the reddish core (**B**).

Complying with one of our previous works [41], the elongational-flow microprocess employed allowed producing monomodal TPGDA/MMA nanodroplets (PDI = 0.08), which were photopolymerized during step 2 to give rise to 227 nm poly(TPGDA-co-MMA) crosslinked nanoparticles (PDI = 0.17). Upon step 3, composite/hybrid polymer microparticles were finally obtained (Figure 5A). They all present a monodispersed size around 800 µm since the coefficient of variation was found to be less than 5%, which complies with a previous study [25]. Each microparticle presents a black/reddish central part, which is attributed to the presence of gold nanoparticles. Indeed it was reported that a solution of about 10 nm gold NPs has a red appearance [42]. Figure 5B shows two individual microparticles, both exhibiting a dense reddish core due to the presence of the gold NPs.

**Figure 6:**
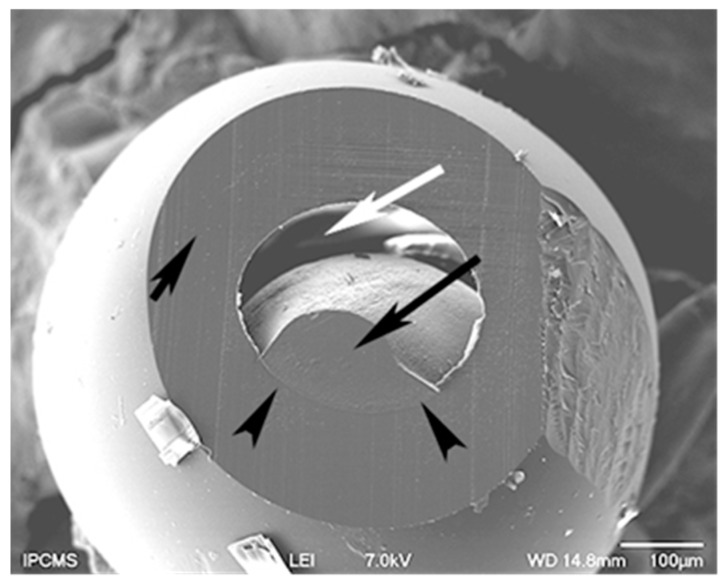
SEM micrograph of a core-shell polymeric microparticle.

A microparticle was ultramicrotomed to unveil the core-shell structure. Figure 6 is a characteristic SEM micrograph. The small black arrow points to the cut shell. It has a thickness around 100 microns. The poly(TPDGA) appears at low magnification like a continuous phase. The long black arrow indicates the cut retracted core, with a limited core-shell interface pointed by the two arrowheads. The white arrow shows the empty space created by the drying process of the microparticle. Indeed, removing the water from the poly(acrylamide) hydrogel induced the core to shrink and as such appears like a hazelnut.

**Figure 7:**
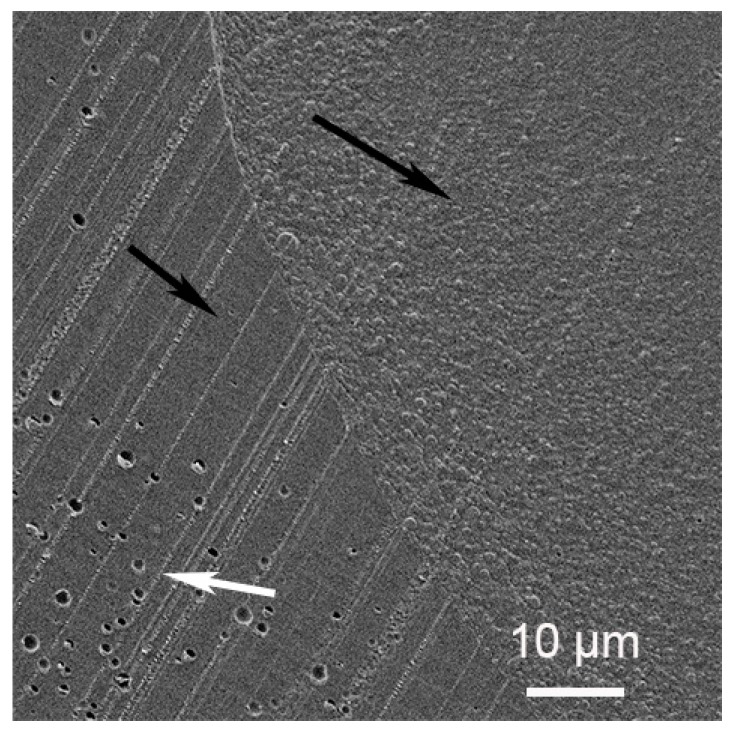
High magnification of the microparticles core-shell interface.

A close look at the interface can be seen at high magnification in Figure 7. The small and long black arrows point to the shell and the core, respectively. The parallel strips are a cutting artifact due to the knife clearly seen in the shell region. Within this region, empty holes are detected (white arrow). They are not seen homogeneously distributed in the shell. We suspect that the aqueous DEG used as a dispersion medium for the silver salt is expelled during the organic TPGDA polymerization process, resulting in drops formation. The drying process has left them empty. The core region appears granular.

**Figure 8:**
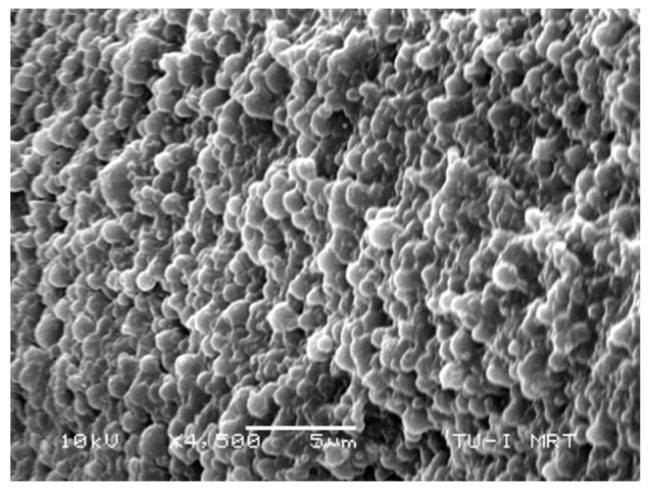
SEM micrograph of the poly(acrylamide) core matrix exhibiting the presence of poly(TPGDA-co-MMA) nanoparticles.

To better characterize it, we have fractured a microparticle and observed the inner core region as shown in Figure 8. It clearly revealed the presence of about 227 nm poly(TPGDA-co-MMA) nanoparticles embedded in the poly(acrylamide) core matrix.

**Figure 9:**
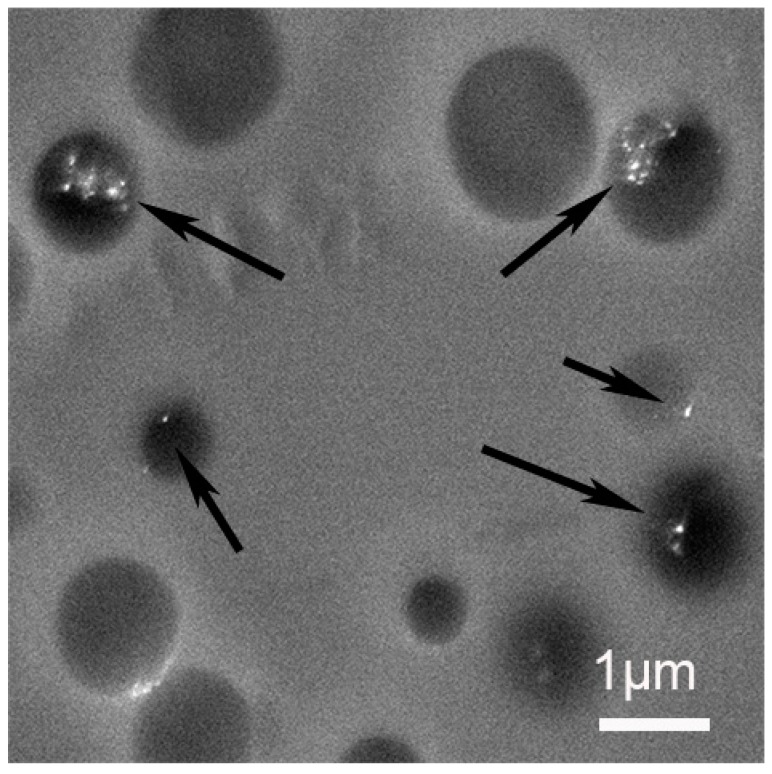
SEM compo image of a shell region recorded at 6 kV.

In Figure 9, the compo image of the shell region shows the presence of metallic nanoparticles (black arrows). The majority of NPs are located in the vicinity of the empty holes. This corroborates our assumption that DEG has been expelled from the organic matrix during polymerization, leading to the presence of NPs in the holes. The nature of those NPs is confirmed by X-rays analysis to be silver (long arrows) or in rare cases gold (short arrows). The unexpected presence of gold NPs inside the shell could be an artifact due to the cutting step.

**Figure 10:**
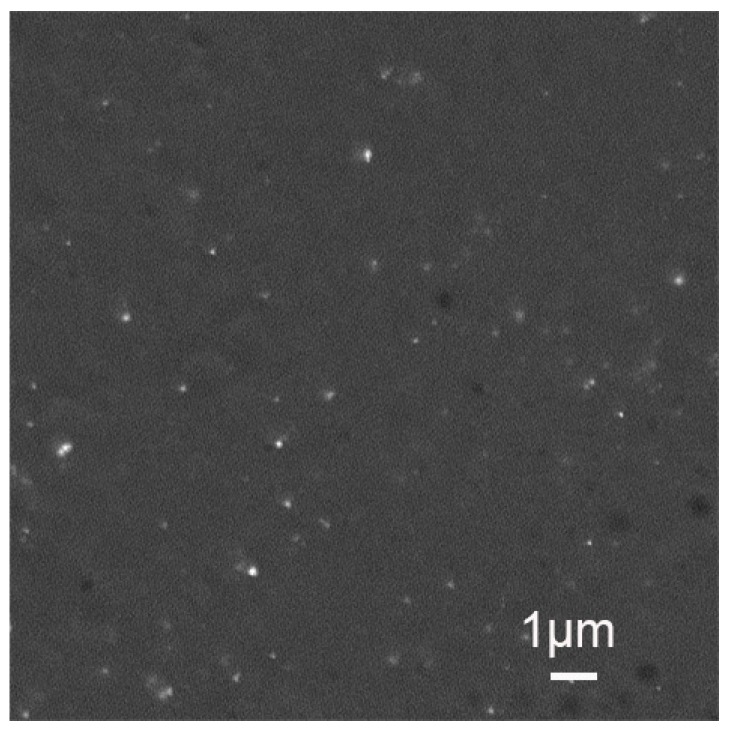
SEM compo image of a core region.

As for the core, the presence of supposedly metallic NPs is confirmed by compo images (Figure 10). Unfortunately, due to the electron beam sensitive nature of the poly(acrylamide) core matrix, we were not able to perform EDX analysis in order to discriminate between gold and silver before melting of the analyzed region. A way to limit this melting process is to lower the energy at 1 kV even though it does not allow the acquisition of an EDX-spectrum but the pure BSE images at 1 kV should give the signal of higher Z-elements like gold. This hope happens to be unreachable as the Au NPs particles are embedded in our case. Moreover, the beam penetration depth at 1 kV is of the order of 2 nm, much too small to detect our gold particles. Indeed, Lu et al. reported that extremely small spherical Au NPs (c.a., 2.5 nm) could be synthesized by photoreduction [42]. However, they also observed that they aggregated into flower-like nanostructures of several tens of nanometers when increasing the irradiation time. Since in this study the photopolymerization was concomitant to the photoreduction, the viscosity of the core phase had probably increased rapidly, which likely had impeded the aggregation of single Au NPs into bigger flower-like nanostructures.

**Figure 11:**
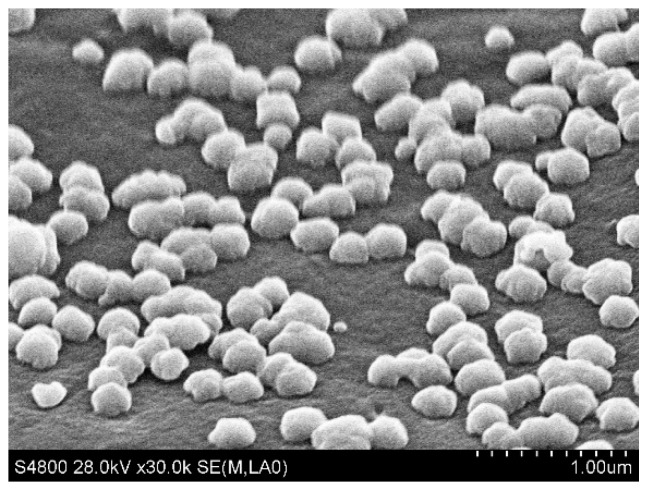
SEM micrograph of the surface of a core-shell polymeric microparticle after silver reinforcement. The silver particles are seen white due to the Z-contrast imaging.

To better confirm the presence of Ag NPs at the microparticles surface, we used a silver reinforcement technique. These NPs served as seeds for the synthesis of Ag^0^ obtained from the reduction of the silver salt by the ascorbic acid. As a consequence, the large Ag nanoparticles (300 nm) to be seen at the surface of the microparticles (Figure 11) testify of the presence of these original Ag NPs produced during the UV-irradiation of the double microdroplets. One can assume that during the droplet formation, DEG and silver salt present in the shell phase (TPGDA) may have migrated toward the outer surface in contact with the aqueous carrier fluid because of their higher affinity with a hydrophilic compound (water) than with a hydrophobic one (TPGDA).

## 4. Applications and Limitations

Such a kind of composite/hybrid multi-scale and multi-domain polymeric microparticles may be used as a multipurpose platform suitable for different applications such as: (i) chemical sensors, a single core-shell microparticle may serve as a substrate for surface enhanced Raman spectroscopy (SERS) detection and quantification of multiples analytes [39] and (ii) carriers for selective multiple-drug release strategies and bioimaging [36,38,43]. The proposed three-step process may present a limitation in term of productivity (i.e., particle generation frequency). Indeed, with the current particle size targeted (800 µm), the last step allowed a production of one double droplet (i.e., one particle) every 3 s. However, by lowering the size down to 400–500 µm and increasing proportionally the three phases flow rate, one can expect to increase the generation frequency to few Hz. Nonetheless, it is worthy to note that due to the extreme sensitivity of SERS, only one particle can be used as a chemical sensor.

## 5. Conclusions

A new facile microfluidic route to the synthesis of core-shell polymeric microparticles doped with organic and inorganic nanoparticles was presented. The approach follows a three-step procedure. First of all, a TPGDA/MMA oil-in-water nanoemulsion was produced in an elongational-flow microprocess. Then the monomers nanodroplets were polymerized by a continuous-flow UV-initiated miniemulsion polymerization process to give rise to a colloidal suspension of polymer nanoparticles. The nanosuspension was then admixed with a water-soluble monomer (acrylamide) and gold salt. Resulting solution composed the core phase of a co-axial capillaries-based microfluidic droplet generator, which produced size-controlled double droplets. The shell phase was composed of a TPGDA, DEG and silver salt mixture. Finally, online UV-irradiation allowed the double droplets polymerization and metal salt reduction. Resulting 800 µm core-shell composite/hybrid polymeric microparticles were extensively characterized by scanning electron microscopy and energy dispersive X-rays spectrometry. Analyses revealed the truly core-shell morphology, the presence of poly(TPGDA-co-MMA) nanoparticles of 227 nm in the poly(acrylamide) core along with supposedly gold nanoparticles, the presence of principally silver particles in the inner and outer surfaces of the poly(TPGDA) shell as well as in spherical regions of supposedly DEG compound inside the shell. After a silver reinforcement procedure, bigger silver nanoparticles (300 nm) were revealed at the surface of the microparticles.
